# School Teachers’ Perceptions of Adolescent Human Papillomavirus (HPV) Vaccination: A Systematic Review

**DOI:** 10.3390/vaccines12040361

**Published:** 2024-03-27

**Authors:** Jihye Choi, Efrat K. Gabay, Paula M. Cuccaro

**Affiliations:** 1 Department of Health Promotion and Behavioral Sciences, School of Public Health, The University of Texas Health Science Center at Houston, 7000 Fannin St., Houston, TX 77030, USA; paula.m.cuccaro@uth.tmc.edu; 2 Center for Health Promotion and Preventive Research, School of Public Health, The University of Texas Health Science Center at Houston, 7000 Fannin St., Houston, TX 77030, USA; efrat.k.gabay@uth.tmc.edu

**Keywords:** teachers, adolescents, HPV, vaccine, recommendations, attitudes

## Abstract

School nurses are uniquely positioned to educate students about immunizations, including human papillomavirus (HPV) vaccination, but schools are often without a nurse for different reasons. In lieu of nurses, teachers who closely interact with students and are traditionally well-trusted by parents may be able to communicate about HPV vaccination, alleviating parental vaccine hesitancy. This systematic review explores school teachers’ perspectives on adolescent HPV vaccination and factors influencing their willingness to make vaccine recommendations. We searched three databases with appropriate medical subject headings and keywords to identify relevant studies. We reviewed fifteen studies and provided an extensive summary and a comparison of the results across the studies. Teachers had low to moderate levels of HPV knowledge with low self-efficacy to counsel parents about the HPV vaccine and expressed concerns about the vaccine condoning adolescent sexual activity, vaccine side effects, and parental disapproval. Nonetheless, some teachers showed interest in learning about vaccine effectiveness in preventing HPV-associated cancers and wanted guidance on vaccine communication with parents, viewing schools as adequate venues to promote and deliver HPV vaccines. Schools should consider educating teachers on HPV and HPV vaccination, with a focus on effective vaccine communication practices to increase adolescent HPV vaccine uptake.

## 1. Introduction

Cancers associated with human papillomavirus (HPV) are vaccine-preventable, and near elimination of such cancers can be achieved with on-time, gender-neutral, HPV vaccination. Increasing evidence indicates that the HPV vaccine is safe and provides a more potent immune response for maximum protection if it is administered at ages 9 to 14 years, before sexual debut [1]. While some countries have achieved promising HPV vaccine coverage, such as Australia, where 80% of female adolescents and 77% of male adolescents have completed the vaccine series in 2022 [2], these rates continue to be suboptimal in many parts of the world. Globally, only about 17% of girls and 5% of boys were fully vaccinated against HPV, and 21% and 7% of girls and boys, respectively, received at least one dose in 2022 [2]. Low uptake of the HPV vaccine is primarily due to parents’ lack of knowledge, negative perceptions of the vaccine, and lack of provider recommendation [3,4,5]. In addition to little understanding of the importance of the HPV vaccine, parents may have the misconception that vaccination equates with permission for early sexual initiation, skepticism around vaccine side effects, and low perceived risk of HPV, which is common among parents of boys due to the persistent overidentification of HPV with females [6].

Schools, where adolescents spend most of their time, are ideal settings for HPV vaccine promotion especially in those staffed with nurses. Bridging the health and education sectors, school nurses are uniquely positioned to improve adolescents’ awareness and understanding of immunizations, including HPV vaccination [7]. School nurse efforts can help parents become well-informed before obtaining vaccination services for their children in clinical settings. However, an ongoing shortage of school nurses worldwide presents a potential gap in providing vaccine recommendations [8,9]. The next decade is likely to reveal a significant school nurse workforce shortage [10], with many countries already experiencing the absence of school nurses [11]. In lieu of school nurses, teachers who regularly and closely interact with students and are traditionally well-trusted by parents may be able to communicate about HPV vaccination to students and parents, thus improving their knowledge of the vaccine and alleviating parental vaccine hesitancy. Given the influential role of teachers, there is value in raising their awareness of the importance of the prophylactic HPV vaccine to facilitate effective vaccine communication in schools.

A plethora of previous research, including a recent review, has examined school nurses’ knowledge and perceptions of adolescent HPV vaccination [12,13,14,15,16,17]. However, fewer studies have engaged school teachers and no review has been conducted to date on this topic. This systematic review aimed to explore the current literature on school teachers’ perspectives on adolescent HPV vaccination and factors influencing teachers’ willingness to make HPV vaccine recommendations to students and parents.

## 2. Methods

A systematic review was conducted and reported using the guidelines of the Preferred Reporting Items for Systematic Reviews and Meta-Analyses (PRISMA) 2020 checklist [18]. A detailed protocol for this review was registered a priori with PROSPERO (CRD42023429812), an international database of prospectively registered systematic reviews. 

### 2.1. Search Strategy

We performed a literature search on three major electronic databases (PubMed, Embase, and Medline OVID) in July 2023. The search strategy comprised three categories of keywords: HPV vaccination, school teachers, and vaccine acceptance. The following medical subject heading (MeSH) terms along with relevant keywords or their variants were included in the advanced search process using the conjunction “AND” and the disjunction “OR”: human papillomavirus, HPV vaccine, HPV vaccination, school teacher, teacher, school staff, knowledge, awareness, attitude, belief, perception, acceptability, intention, and refusal. Reference lists of eligible articles were also manually checked to retrieve other potentially relevant articles and all “related to” or similar articles of the identified articles were followed. 

### 2.2. Inclusion and Exclusion Criteria

This review included peer-reviewed articles in the scientific literature that constituted original research. To understand recent research trends, articles had to be published within the last decade between 1 January 2013 and 30 June 2023, and the publication language was restricted to English. The review included articles that specifically reported on school teachers’ and staff’s knowledge and perceptions of adolescent HPV vaccination, willingness to recommend the vaccine to students and parents, and perspectives on HPV vaccine delivery or promotion in school settings. No restriction was placed on the type of school or geographic region. Articles were excluded from the review if they focused on only school nurses or teachers’ HPV vaccination status and personal determinants of vaccination without any reference to their attitudes towards student vaccination. Articles were also excluded if they were systematic or scoping reviews, meta-analyses, project protocols, conference proceedings, briefing reports, or publications from non-indexed journals. 

### 2.3. Selection Process and Data Synthesis

Upon obtaining the search results, all duplicated articles across the databases were removed using Endnote, followed by a manual verification. Titles and abstracts of the remaining articles were independently screened by the authors against the inclusion and exclusion criteria. Articles of the selected abstracts were retrieved and full-text reviewed. Articles were eliminated if their full-text versions were not available. Full-text reviewed articles that adhered to the eligibility criteria were included in the final synthesis and qualitatively analyzed. This qualitative analysis entailed an extensive summary of the eligible studies and a risk of bias assessment of their methodological quality, followed by a comparison of the results across the studies.

### 2.4. Quality Assessment

The methodological quality and risk of bias for the included studies were appraised using three validated instruments. The quality of the quantitative studies was assessed using the 7-item Joanna Briggs Institute (JBI) Critical Appraisal Checklist with three response options (yes, no, unclear) [19]. The qualitative studies were assessed on the level of risk of bias (i.e., low risk, high risk, or unclear) using the 10-item Critical Appraisal Skills Programme (CASP) Checklist [20]. The quality of the mixed-methods studies was assessed using the 5-item Mixed Methods Appraisal Tool (MMAT) with three response options (yes, no, can’t tell) [21]. An overview of the three assessment tools is included in Supplementary Table S1. 

## 3. Results

### 3.1. Search Results

**Figure 1:**
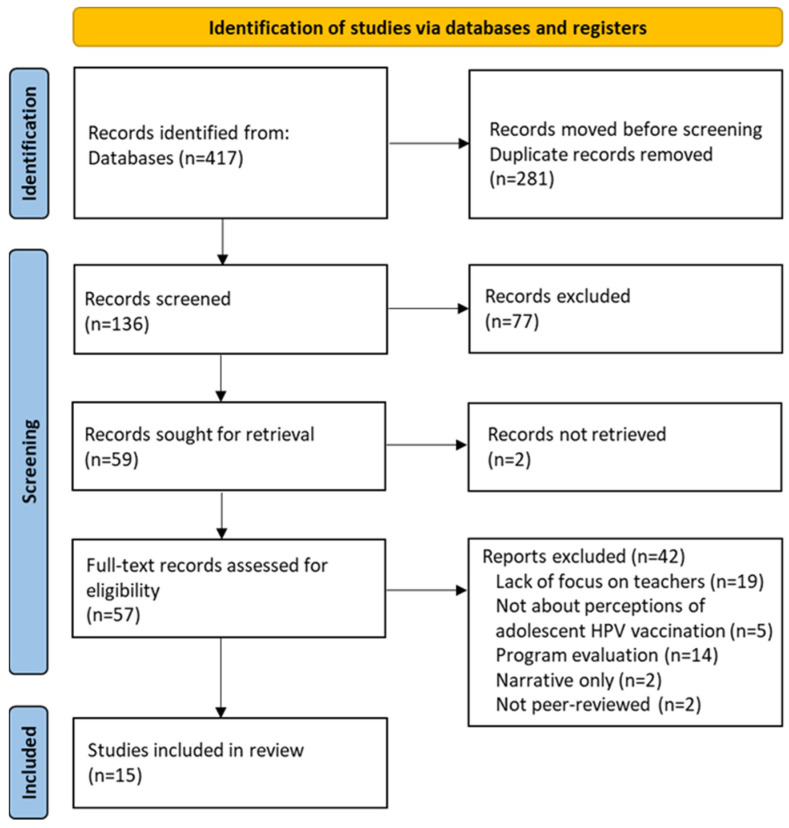
PRISMA flow diagram.

The literature search and review process are shown in Figure 1. After the removal of duplicates within and across databases (Embase, Medline, and PubMed), titles and abstracts of 136 unique articles were screened to identify whether they adhered to the eligibility criteria and qualified for full-text review. We full-text reviewed 59 articles and excluded 44 articles for the following reasons: lack of focus on school teachers (n = 19), not focused on perceptions of adolescent HPV vaccination (n = 5), unavailability of full-text article (n = 2), focus on vaccination program evaluation (n = 14), narrative only (n = 2), and not peer-reviewed (n = 2). We selected a total of 15 studies published between 1 January 2013 and 30 June 2023 as the final set of records for the review. No additional eligible articles were identified. 

### 3.2. Study Characteristics

Table 1 shows the characteristics of the included studies [22,23,24,25,26,27,28,29,30,31,32,33,34,35,36]. Studies were conducted in various geographic settings: five studies from Asia (Hong Kong, Japan, the Republic of Korea, and Uzbekistan), two studies from North and South America (Canada and Peru), five studies from Africa (Kenya, Nigeria, and Tanzania), and three studies from Europe (France and Scotland). Five studies focused on teachers from elementary/primary schools, six studies involved teachers from secondary schools (middle or high schools), two studies included teachers from both primary and secondary schools, and two studies did not specify the type of school for their participants. Four studies specified the subject taught by the teachers. As for primary study outcomes, seven studies examined teachers’ support for and acceptance of adolescent HPV vaccination, five studies assessed their willingness to communicate and recommend the HPV vaccine to students and parents, and three studies included both acceptance of and willingness to recommend the vaccine. Factors affecting these primary outcomes were categorized as either barriers or drivers of teachers’ acceptance of adolescent HPV vaccination. Barriers were teachers’ lack of HPV knowledge, negative vaccine attitudes (low perceived need for the HPV vaccine, distrust towards the HPV vaccine, and perceived burden of HPV vaccine promotion), and fear of parents’ HPV vaccine disapproval. Drivers were teachers’ perceived benefits of adolescent HPV vaccination, HPV awareness and desire for more HPV education, and perception of schools and teachers as important avenues for HPV vaccine promotion. Table 2 shows a detailed summary of each study.

### 3.3. Quality Assessment of the Included Studies

**Figure 2:**
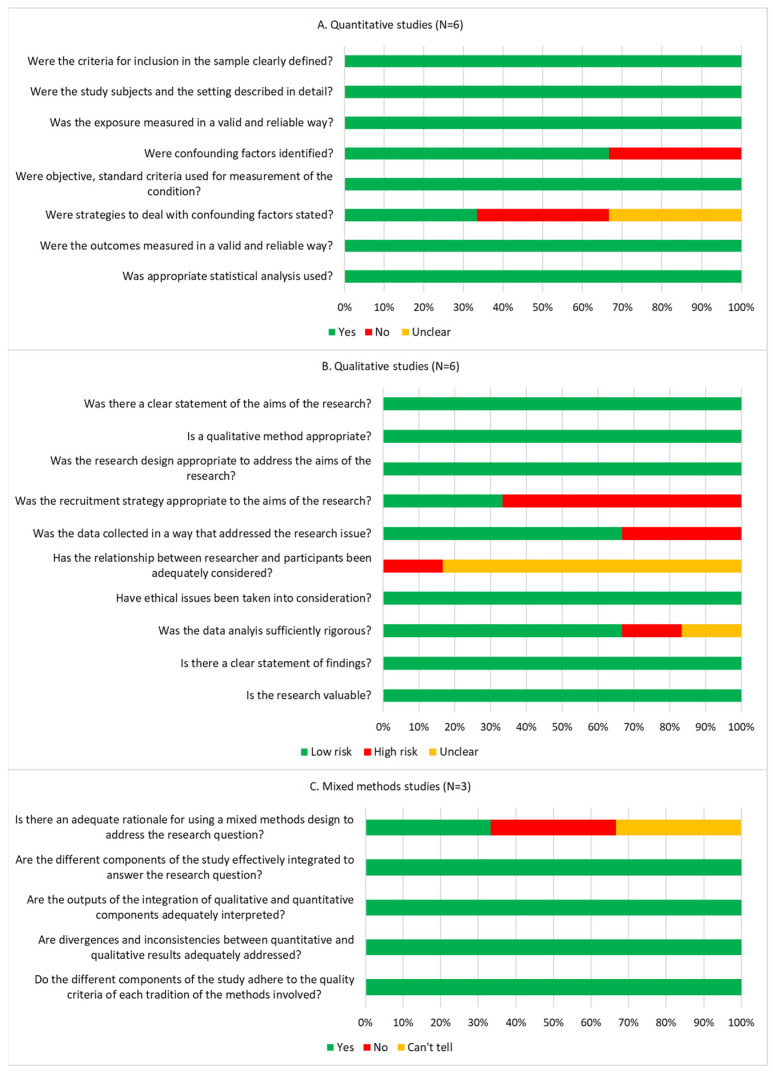
Quality assessment of the included studies.

All six quantitative studies [22,24,25,26,31,35] provided a detailed description of their study setting and participants, measured variables of interest in a valid and reliable way, and used appropriate statistical analyses. Non-compliance to quality criteria for the quantitative studies was mostly due to limited or unclear reporting of confounding factors and how they were managed in the analysis. All six qualitative studies [23,28,29,30,33,36] stated the research aims, used appropriate qualitative methods, reported ethics approval, and clearly explained the value of their findings. Four studies [23,28,29,33] did not adequately address the recruitment strategy and had minor concerns about the small sample size, which may have incurred selection bias. Five studies [23,28,29,33,36] had unclear reporting of whether the relationship between the researcher and participants had been adequately considered during the interviews. All three mixed-methods studies [27,32,34] were compliant with the quality criteria except for the item regarding adequate rationale for using a mixed-methods design to address the research question (only one study [27] met this criterion). Overall, the included studies were relevant to the topic, presented their data and research findings coherently, and reflected on their methodological limitations. Figure 2 illustrates the quality assessment of the included studies in detail.

### 3.4. Synthesis of Evidence

#### 3.4.1. Lack of Knowledge

Ten studies [25,27,28,29,31,32,33,34,35,36] described teachers’ overall lack of knowledge regarding the etiology of HPV-associated diseases, the availability of the HPV vaccine and its effectiveness in preventing HPV-associated cancers, and boys’ eligibility for HPV vaccination. Less than 50% of primary, middle, and high school teachers in six studies conducted in Nigeria, Kenya, Japan, Tanzania, and France knew that HPV causes genital warts and cancer and had heard of the HPV vaccine and its benefits in preventing cervical cancer [25,27,31,32,34,35]. Less than 5% of teachers in Kenya knew that the vaccine can prevent vulvar and anal cancers in addition to cervical cancer [32], and 23% of teachers in a French study responded correctly that HPV can cause oral cancer [27]. In one qualitative study from Kenya, when teachers were asked about the causes of cervical cancer, HPV was rarely mentioned as a primary cause among many possibilities, such as not practicing self-hygiene [33]. Two qualitative studies from Hong Kong and Uzbekistan provided evidence that teachers across all school levels lacked knowledge regarding the HPV vaccine and who should receive it, as well as vaccine safety, which led to their vaccine hesitancy [29,36]. The disconnect between HPV risk and males was clear. For example, 8% and 71% of primary and middle school teachers in Kenya and France, respectively, answered correctly that HPV infects both men and women [27,32]. Another qualitative study of middle school teachers in France, a country that promoted gender-neutral HPV vaccination, reported that teachers regarded HPV as an infection concerning females and was irrelevant to males, commonly labeling the vaccine as the “cervical cancer vaccine” [28]. At least one teacher in each of the four focus group studies [27,28,32,34] questioned the importance or benefits of the HPV vaccine for boys. Lack of knowledge about HPV and HPV vaccination was observed among teachers regardless of the grade levels they served in a mix of high- and lower-income regions. 

#### 3.4.2. Negative Attitudes towards HPV Vaccines 

Twelve studies reported on teachers’ negative attitudes towards promoting HPV vaccination to their students [22,23,24,25,26,27,28,29,30,32,33,36]. Six studies from the Republic of Korea, Kenya, Hong Kong, and France delineated low perceived susceptibility to HPV as reasons for teachers’ non-acceptance of HPV vaccination [22,27,28,29,32,33]. HPV vaccination was not prioritized compared to other health education topics, such as influenza vaccination, especially at the primary school level, based on the belief that students were too young to be considered vulnerable to sexually transmitted diseases [27,28,29,33]. The concept of preventing cervical cancer with a vaccine associated with risky sexual behavior was perceived to be superfluous and difficult to understand for young students. Teachers also demurred from providing younger students with “too much” information about sexual health [23]. Distrust towards the HPV vaccine was common among teachers who claimed that insufficient research has been conducted on the vaccine and who were concerned about vaccine side effects [25,26,30,32,33,36]. In two qualitative studies from Peru and Uzbekistan, primary school teachers believed that the HPV vaccine may harm girls’ reproductive health and future fertility and would first wait to see how others fared before making HPV vaccine recommendations [30,36]. In another qualitative study from Kenya, in which the school level was not reported, teachers had mixed views pertaining to fertility; while protecting a girl’s fertility was a driver for HPV vaccine acceptance, the same vaccine generated fear in terms of harming the girl’s fertility [33]. Four studies from the Republic of Korea, Scotland, Kenya, and Hong Kong reported on teachers’ concerns about seemingly condoning or encouraging adolescents’ sexual promiscuity by promoting the vaccine [22,23,29,32]. They expressed discomfort that early HPV vaccination could potentially compromise students’ childhood innocence and that students would misinterpret the vaccine as permission for premarital sexual initiation [23,29]. In one study conducted in France, sexuality being a cultural taboo made it difficult for school staff to discuss sexually transmitted infections in class [28]. Teachers from the Republic of Korea, Scotland, Kenya, France, and Uzbekistan were also reluctant to promote HPV-related prevention behaviors because they saw health and education as separate silos; across all school levels, HPV vaccine promotion was not considered teachers’ responsibility but rather seen as a substantial burden added to their work [22,23,27,28,33,36]. 

#### 3.4.3. Fear of Parents’ HPV Vaccine Disapproval

Teachers identified anticipated negative reactions from parents as a significant barrier to discussing the HPV vaccine with parents due to its association with sexuality, which hindered teachers’ willingness to make vaccine recommendations [23,24,27,28,29,30,33,34,36]. One qualitative study of primary and middle school teachers in Hong Kong articulated that parents are the predominant partners of schools according to the home-school-doctor model, and teachers could not justify HPV vaccine promotion in school settings without the support of parents [29]. Given the possible controversy around HPV vaccination, teachers across school levels from Scotland, Uzbekistan, and France feared parental complaints if they were seen to promote the vaccine and did not want to be held responsible by parents for any potentially adverse health outcomes in children due to vaccination [23,27,28,36]. Qualitative studies of primary and middle school teachers from Hong Kong and Peru found that parents’ lack of knowledge and interest in HPV vaccination as well as their disapproval of the vaccine diminished teachers’ self-efficacy and willingness to recommend the vaccine to students [29,30]. Teachers in a Canadian study also reported low levels of confidence (M = 2.8/7) in discussing HPV vaccination with parents of school-aged children [24]. Convincing parents to vaccinate their child against HPV was challenging for teachers if parents did not believe in scientific evidence for HPV vaccine efficacy and if teachers themselves did not have formal education about the vaccine. To that end, a qualitative study from Kenya found that parents’ persistent mistaken beliefs that HPV vaccination encourages sexual initiation among young children dampened teachers’ willingness to communicate about the vaccine [33]. Similar to the other barriers described in this review, teachers’ fear of parental vaccine disapproval was documented in studies from regions with varying degrees of income levels.

#### 3.4.4. Drivers of Teachers’ Acceptance of Adolescent HPV Vaccination 

Despite low HPV knowledge and skepticism towards the HPV vaccine—variables most dominant across the studies—teachers across school levels expressed interest in knowing about HPV and HPV vaccination before making vaccine recommendations. In seven studies from the Republic of Korea, Japan, Kenya, Tanzania, Canada, and France, teachers across varying school levels demonstrated positive attitudes towards HPV vaccine promotion as they sought more education about HPV and the vaccine, coupled with accurate and up-to-date information [22,24,25,28,32,34,36]. For example, 89.1% of Korean teachers expressed a desire to know more about HPV and the HPV vaccine [22]. The information that Canadian, Japanese, and Tanzanian teachers most wanted to obtain was about HPV vaccination in males, long-term vaccine side effects, and proof of the vaccine’s preventive effect [24,25,34]. Two studies from Canada and Japan, in which HPV education was provided to teachers as part of their research, found that willingness to recommend the vaccine significantly improved following the educational intervention compared to their baseline results (*p* < 0.05) [24,26]. Knowing that parents place great trust in them, teachers were willing to take on a role in communicating with parents about the HPV vaccine. For example, two qualitative studies of primary school teachers from Peru and Uzbekistan requested guidance on effective communication with parents about HPV vaccination and cancer prevention [30,36]. Finally, in five studies from Kenya, Tanzania, Nigeria, and France, primary and middle school teachers acknowledged that schools are appropriate venues for the education, promotion, and delivery of HPV vaccination given their daily contact with children [27,28,33,34,35]. 

## 4. Discussion

The growing recognition of the global scarcity of school nurses has shed light on school teachers’ potential to provide students and parents with HPV vaccine recommendations. This systematic review revealed that while there was an inclination to accept and promote adolescent HPV vaccination, teachers confronted predominantly barriers to making HPV vaccine recommendations. These barriers can be mitigated by implementing educational interventions and providing tailored vaccine communication training to increase teachers’ HPV knowledge and self-efficacy for HPV vaccine communication. 

The lack of knowledge about HPV and HPV vaccination among teachers is not surprising given that explicit vaccination education is not part of their employment mandate [37]. In the absence of school nurses in low-resource schools, however, teachers may be required to serve overlapping roles in the classroom as educators and health managers [38]. These circumstances indicate the need for teachers’ increased access to health-related information, including HPV immunization. Previous research found that school staff were not aware of specifically the prevalence and age distribution of HPV infection [39]. Our results show teachers across school levels, especially those in African regions, have a limited understanding of the connection between HPV and noncervical HPV-associated cancers and the effectiveness of HPV vaccination. They seemed to heavily focus on the prevention of cervical cancer compared with other HPV-associated cancers, such as oral, anal, and oropharyngeal cancers. The mere focus on cervical cancer prevention in countries that have yet to adopt gender-neutral HPV vaccination may have hindered teachers from recognizing the necessity of vaccinating boys, despite the growing burden of noncervical HPV-associated cancers for males [40]. In fact, studies in this review were conducted in countries without gender-neutral HPV vaccination at the time of publication, except for Canada, France, and Scotland, although French teachers had little awareness of male eligibility for HPV vaccination [27,28]. School districts and healthcare providers should consider investing in concerted efforts to provide teachers with short, web-based continuing education to maximize reach and improve content knowledge [41]. This education should be designed to inform teachers about HPV consequences to increase the perceived severity of HPV and defeminize HPV by highlighting the importance of vaccinating all genders. Importantly, primary teachers from three different regions all worried that the HPV vaccine may harm fertility as its side effect [30,33,36], a concern often raised by caregivers [42]. Therefore, educators should prioritize providing accurate, evidence-based information pertaining to vaccine side effects to allay distrust towards HPV vaccine safety for teachers of younger children.

Health problems and risk-taking behavior such as unsafe sexual activity are associated with low scholastic performance in adolescents [43]. However, it was evident that teachers tend to perceive health and education as separate silos and that becoming embroiled in HPV vaccine promotion would equate to unwanted additional work. For example, two studies from France and Uzbekistan conveyed teachers’ argument that HPV vaccine promotion should involve health workers and general practitioners, rather than school staff, in which case parents would be more receptive and consider vaccinating their children [27,36]. These perceptions deterred teachers’ willingness to make vaccine recommendations. Additional professional development may be necessary to inculcate teachers with their role in helping students safely transition into adulthood, and the value in ensuring students’ academic achievement as well as primary prevention practices. For teachers, recognizing themselves as influential figures in protecting adolescents from adverse behavioral outcomes may be a stronger priority predictor of making HPV vaccine recommendations than having HPV knowledge [15]. School-based vaccination is considered the most efficient means of reaching high vaccine coverage for adolescents, especially given reduced visits to healthcare providers as youth transition from childhood to adolescence [44]. As earlier studies have emphasized the effective use of schools for HPV vaccine programs in successfully adopting HPV vaccination in low-resource settings, our review also confirmed that teachers from various geographic regions, regardless of the grade levels they serve, view schools as an appropriate venue for the education, promotion, and delivery of HPV vaccines. Teachers’ sensitization to HPV and adequate levels of commitment and engagement in school-based HPV vaccination programs are important facilitators of vaccine uptake among students [37,45]. 

Finally, we observed that teachers were less willing to recommend the HPV vaccine due to its association with sexually transmitted diseases and concerns about negative reactions from parents. Initiating conversations about HPV vaccination becomes even more difficult for teachers when parents are reluctant about the vaccine based on unverified assumptions. Teachers working in schools that implement school-based HPV vaccination programs have reported typically not feeling comfortable about the vaccine or promoting its use [46]. In another study, school staff appeared disinclined to accept adolescent HPV vaccination because of concerns about generating antagonism between parents and the school [47]. Although initial challenges may arise, enlisting school teachers can be a promising strategy to attenuate parents’ vaccine hesitancy and promote the less-recognized HPV vaccine. Teachers’ attitudes will be crucial, especially when discussing difficult subjects with parents [48]. In this review, almost half of the studies (46.7%) affirmed teachers’ desires for additional HPV information and guidance on effective communication with parents regarding the vaccine, for which they had low self-confidence. A “train-the-trainer” approach may be a feasible option, where teachers are formally trained on how to deliver HPV vaccine recommendations and address caregivers’ questions and concerns about vaccine safety using research-tested messages [49]. A recent US study on increasing adolescent HPV vaccination corroborated that healthcare providers’ attendance in training on counseling hesitant parents was associated with increased motivation to routinely recommend the vaccine as well as increased positive vaccine attitudes and self-efficacy, which also led to a small increase in adolescents’ vaccine uptake [50]. Other studies have noted that formal scientific training was associated with increased vaccine confidence among teachers [37,51]. These findings suggest that a public health partnership opportunity with teachers may be successful in increasing not only their vaccine confidence but also their self-efficacy for vaccine communication.

### Strengths and Limitations

This review has a few limitations. First, we excluded studies that mainly discussed the design and implementation of school-based or community-based HPV vaccination programs, but such studies may have assessed vaccine attitudes of school staff. Second, imposing a restriction on the publication year may have limited inclusion of other relevant research conducted outside of the specified publication years. The rationale for choosing a ten-year window was to stay current with the growing HPV literature and shifts in HPV vaccination considerations regarding gender, schedules, and recommendations. Lastly, the findings of this review may not be applicable to private or religiously affiliated schools and schools in all countries, especially the US, as no study from the US was included. The significance of our review is that it provides insights into teachers’ perceptions of adolescent HPV vaccination compared to other reviews that have focused on school nurses. Another strength is that this review presents data from low-, middle-, and high-income countries. Schools can refer to this review to prepare training that addresses teachers’ concerns and equips them with the needed knowledge before implementing school-based HPV vaccination programs.

## 5. Conclusions

This systematic review highlights school teachers’ perspectives on adolescent HPV vaccination and factors influencing their willingness to recommend the vaccine to students and parents. We observed that some teachers were cognizant of the importance of HPV vaccination and held positive vaccine attitudes. However, overall lack of HPV knowledge, skepticism of the HPV vaccine, and fear of disapproval from parents were substantial barriers to teachers’ willingness to recommend the vaccine to students and parents. These findings suggest that schools, especially those without nurses, should seek opportunities to offer teachers education on HPV and HPV vaccination as well as formal training on HPV vaccine communication practices on the road to increased HPV vaccine uptake among adolescents, both females and males.

## Figures and Tables

**Table 1:** Characteristics of the included studies, 2013–2023 (n = 15).

Study Characteristics		n (%)
Region	Asia	5 (33.3%)
	North America	1 (6.7%)
	South America	1 (6.7%)
	Africa	5 (33.3%)
	Europe	3 (20.0%)
Region income level	High	7 (46.7%)
	Upper-middle	2 (13.3%)
	Lower-middle	6 (40.0%)
School level	Elementary/primary school	5 (33.3%)
	Secondary (middle/high) school	6 (40.0%)
	Both primary and secondary school	2 (13.3%)
	Not specified	2 (13.3%)
Teacher type	Health/Health Sciences	2 (13.3%)
	Sciences/Life Sciences	1 (6.7%)
	Arts and Math	1 (6.7%)
	Not specified	11 (73.3%)
Study design	Quantitative	6 (40.0%)
	Qualitative	6 (40.0%)
	Mixed methods	3 (20.0%)
Primary outcome measure	Acceptance of HPV vaccination	7 (46.7%)
	Willingness to recommend HPV vaccination	5 (33.3%)
	Both acceptance of and willingness to recommend HPV vaccination	3 (20.0%)

**Table 2:** Summary of the included studies.

Study	Aim	Country	Study Design and Size	School Level	Gender-Neutral HPV Vaccination ǂ	Key Findings
High income region †						
Choi et al., 2013 [22]	To identify factors associated with Korean health teachers’ intention to recommend the HPV vaccine	Republic of Korea	Quant N = 119	Elementary, Middle, High	No, female-only	Less than 12% of teachers reported having recommended HPV vaccination to students and parents. The mean score of the intention to recommend the HPV vaccine was 5.29 out of 10. Teachers had the highest intention to recommend the vaccine to high school students or their parents (6.12), followed by middle school students or their parents (5.32) and elementary school students or their parents (4.45). Teachers did not consider themselves responsible for promoting the vaccine due to having a heavy workload. *
Spratt et al., 2013 [23]	To examine secondary school teachers’ views of their roles as partners in a school-based HPV vaccination program	Scotland	Qual N = 32	Secondary	Yes	Teachers were concerned about the impact of vaccination on students’ current understanding of sex and sexuality. They showed unease that vaccination could potentially compromise childhood innocence. Some teachers feared negative publicity or parental complaints if they were seen to promote adolescent HPV vaccination. *
Rosberger et al., 2014 [24]	To explore the effect of a workshop intervention designed to provide the most up-to-date information among educators and counselors about their knowledge, attitudes, and beliefs about HPV and the HPV vaccine	Canada	Quant N = 37	Not stated	Yes	Most teachers knew that HPV is sexually transmitted (86.5%) and that the HPV vaccine prevents cervical cancer (83.8%). Teachers reported low levels of confidence (M = 2.8/7) in discussing HPV vaccination with parents. Willingness to recommend the HPV vaccine was not significantly associated with knowledge nor confidence in providing accurate HPV vaccine information. Common types of additional information requested were regarding HPV vaccination in males and the long-term side effects of the vaccine.
Kamada et al., 2018 [25]	To determine the ways to increase teachers’ willingness to encourage the use of the HPV vaccine	Japan	Quant N = 247	Not stated	No, female-only	While 63% knew that the HPV vaccine prevents cervical cancer, 36% knew that HPV causes cervical cancer. Seventy-seven percent of the teachers feared vaccine side effects and 69% would not recommend the vaccine to their daughters and students. The information they most wanted was a proof of the HPV vaccine’s preventive effect.
Ishiwada et al., 2020 [26]	To identify the current status, issues, and barriers regarding HPV vaccination among health science teachers	Japan	Quant N = 37	High	No, female-only	Teachers were initially uncertain (51.3%) and fearful (30.8%) about HPV vaccination. Teachers were significantly more inclined to recommend the HPV vaccine to students (*p* < 0.05) once they were more informed about HPV and became less fearful of HPV vaccine side effects.
Bocquier et al., 2023 [27]	To identify barriers, facilitators, and needs of the different school professionals involved in the implementation of HPV vaccination promotion interventions in French middle schools	France	Mixed N = 315	Middle (94% public schools, 5% private schools)	Yes	Eighty percent of teachers knew that HPV is sexually transmitted, but less than half knew that HPV can cause genital warts, and oral and cervical cancers. Seventy-six percent knew that the HPV vaccine protects against HPV-related cancers, and 56% knew that the vaccine is recommended for boys. Teachers had positive attitudes towards the benefits of HPV vaccination (mean score > 5 on a scale of 1–7). Teachers had mixed views about providing HPV education at school; focus groups agreed that offering HPV vaccination does not fall within the school’s role. Perceived barriers included teachers’ additional workload and fear of parents’ negative reactions.
Ailloud et al., 2023 [28]	To evaluate knowledge, perceptions, beliefs, facilitators, and barriers to HPV vaccination among school staff from middle schools	France	Qual N = 14	Middle	Yes	Teachers lacked HPV knowledge and saw HPV as a women’s issue. Teachers considered that children are too young to receive a sexually-related vaccine. HPV discussion in school was hindered because of sexuality being a taboo and a difficult topic for school staff. Some teachers believed that teachers are a legitimate means to conduct awareness sessions on HPV but felt burdened to do so at the same time. Teachers mentioned that the role of schools could be more important in transmitting information on HPV to students and parents.
Upper-middle income region †						
Siu et al., 2019 [29]	To investigate how school teachers in primary and secondary schools perceive HPV and HPV vaccines	Hong Kong	Qual N = 35	Primary, secondary	No, female-only	Teachers believed that cervical cancer protection and HPV vaccination were difficult concepts for their students who were too young to be considered vulnerable. Schools would oppose HPV vaccine promotion, and it was not prioritized compared to other health education topics (e.g., influenza). Teachers worried that HPV vaccine promotion could convey a negative message on sex attitudes. Parents’ attitudes affected teachers’ motivation. Without parental support, teachers could not justify school-based HPV vaccine promotion.
Llavall et al., 2021 [30]	To understand teachers’ perceived barriers and facilitators to implementing HPV vaccination program, HPV knowledge and attitudes, and recommendations on strategies to increase vaccination rates	Peru	Qual N = 10	Primary	No, female-only	While teachers pointed out a necessity for their students to be protected against cervical cancer, there was distrust towards the HPV vaccine and fear generated in terms of harming adolescents’ fertility. Teachers thought parents were not informed about HPV and the vaccine. Teachers also reported that parents rejected the vaccine because it would lead to sexual initiation among children. Teachers reported perceived parents’ fear of serious side effects such as infertility.
Lower-middle income region †						
Ajah et al., 2015 [31]	To describe the knowledge and attitude of secondary school teachers towards HPV vaccination; to explore the feasibility of enlisting teachers towards promoting vaccine uptake	Nigeria	Quant N = 412	Middle, High	No, female-only	About 80% of teachers who were aware of cervical cancer knew that HPV caused cervical cancer. Among these, less than 40% knew the availability and benefits of the HPV vaccine, and 70% were willing to accept and recommend the vaccine to their daughters and students. Knowledge was significantly associated with HPV vaccine acceptability.
Masika et al., 2015 [32]	To assess primary school teachers’ knowledge and acceptability of HPV vaccine	Kenya	Mixed N = 339	Primary (34 public schools, 3 private schools)	No, female-only	Teachers had low to moderate levels of knowledge about HPV and the HPV vaccine (mean score of 48%), especially men’s susceptibility to HPV infection (mean score of 8%). However, vaccine acceptability was high (89%). One-third of all teachers indicated insufficient vaccine information and fear of vaccine side effects as the main barriers. Nearly all respondents (98%) expressed interest to know more about the HPV vaccine, and 93% supported school-based vaccine delivery.
Vermandere et al., 2015 [33]	To verify teachers’ awareness of and support for HPV vaccination programs; to assess barriers in HPV vaccine promotion	Kenya	Qual N = 43	Not stated	No, female-only	When asked about causes of cervical cancer, HPV was rarely mentioned as a primary cause. Teachers showed distrust towards the HPV vaccine. While protecting a girl’s fertility was a driver for HPV vaccine acceptance, the same vaccine generated fear in terms of harming the girl’s fertility. At least three teachers described perceived parental fear that vaccination would enhance sexual activity among children. Some were keen to provide information and promote the vaccine given their daily contact with the children. *
Keehn et al., 2021 [34]	To assess primary school teachers as key informants when assessing barriers to parent acceptance of the HPV vaccine	Tanzania	Mixed N = 155	Primary	No, female-only	While 95% had heard of cervical cancer, only 37% and 29% of participants had heard of HPV and the HPV vaccine, respectively. Teachers from all seven schools included in this study mentioned parental lack of HPV knolwedge as the main barrier but were willing to promote the vaccine to parents. Common questions from focus groups included: inquiries about vaccine side effects and why boys are not being vaccinated at this time.
Enebe et al., 2021 [35]	To determine the level of awareness, acceptability and uptake of HPV vaccine among female secondary school teachers	Nigeria	Quant N = 377	Secondary	No, female-only	Less than half (41.9%) of the teachers had high knowledge of cervical cancer, and 48.3% knew that HPV vaccination can prevent cervical cancer. Only 14.6% indicated having taught their students about cervical cancer or HPV vaccine. Acceptability was high among teachers who were aware of the vaccine, as the majority of teachers (93.6%) would recommend the vaccine to their children and students if the vaccine were given for free by the government.
Warsi et al., 2023 [36]	To understand barriers and drivers to general and HPV vaccination among key target groups (teachers) in Uzbekistan	Uzbekistan	Qual N = 32	Elementary	No, female-only	Teachers’ vaccine hesitancy stemmed from knowledge gaps on vaccine safety. Few participants were aware of HPV, its relation to cervical cancer, and the HPV vaccine.* The primary anxieties of the teachers were any potential negative effects of the vaccine on students’ future fertility. Teachers highlighted the need for clear and credible information on the safety of the HPV vaccine to be confident in their support for the vaccine.

† Source: The World Bank, using gross national income (GNI) per capita data in U.S. dollars, converted from local currency using the World Bank Atlas method. ǂ Federal approval for gender-neutral HPV vaccination at the time of publication. * Data were not quantified.

## Data Availability

No new data were created or analyzed in this study. Data sharing is not applicable to this article.

## Supplementary Materials

The following supporting information can be downloaded at: https://www.mdpi.com/article/10.3390/vaccines12040361/s1, Table S1: Overview of the quality assessment tools.
