# Supplementary material for: School Teachers’ Perceptions of Adolescent Human Papillomavirus (HPV) Vaccination: A Systematic Review
Source: Vaccines (Basel). 2024 Mar 27;12(4):361. doi: 10.3390/vaccines12040361 (PMC11053788; doi:10.3390/vaccines12040361)
Supplement: Supplementary file 1 [file vaccines-12-00361-s001.zip › vaccines-2890677-supplementary.pdf]

vaccines

MDPI

Supplementary Material

Table S1. Overview of the quality assessment tools.

Study design	Assessment tool	Items	Rating
Quantitative	JBI Checklist	1. Were the criteria for inclusion in the sample clearly defined?<br>2. Were the study subjects and the setting described in detail?<br>3. Was the exposure measured in a valid and reliable way?<br>4. Were objective, standard criteria used for measurement of the condition?<br>5. Were confounding factors identified?<br>6. Were strategies to deal with confounding factors stated?<br>7. Were the outcomes measured in a valid and reliable way?<br>8. Was appropriate statistical analysis used?	Yes<br>No<br>Unclear
Qualitative	CASP Checklist	1. Was there a clear statement of the aims of the research?<br>2. Is a qualitative methodology appropriate?<br>3. Was the research design appropriate to address the aims of the research?<br>4. Was the recruitment strategy appropriate to the aims of the research?<br>5. Was the data collected in a way that addressed the research issue?<br>6. Has the relationship between researcher and participants been adequately considered?<br>7. Have ethical issues been taken into consideration?<br>8. Was the data analysis sufficiently rigorous?<br>9. Is there a clear statement of findings?<br>10. How valuable is the research	Low risk<br>High risk<br>Unclear
Mixed methods	MMAT	1. Is there an adequate rationale for using a mixed methods design to address the research question?<br>2. Are the different components of the study effectively integrated to answer the research question?<br>3. Are the outputs of the integration of qualitative and quantitative components adequately interpreted?<br>4. Are divergences and inconsistencies between quantitative and qualitative results adequately addressed?<br>5. Do the different components of the study adhere to the quality criteria of each tradition of the methods involved?	Yes<br>No<br>Can’t tell
